# Investigation of structural, optical, morphological, photoluminescence and antimicrobial properties of SrAl_2_O_4_:Eu^2+^ nanophosphor by using urea fuel combustion method

**DOI:** 10.1038/s41598-023-29241-4

**Published:** 2023-02-07

**Authors:** Praveen Kumar Litoriya, Swati Kurmi, Ashish Verma

**Affiliations:** Department of Physics, Dr. Harisingh Gour Vishwavidyalaya (A Central University), Sagar, Madhya Pradesh 470003 India

**Keywords:** Chemistry, Materials science, Nanoscience and technology

## Abstract

In the present study, the Sr_1−x_Al_2_O_4_:Eu_x_ (x = 0.00, 0.01, 0.03, 0.05, 0.07, and 0.09) phosphor were synthesized by urea fuel combustion method at 580 °C temperature with very high brightness and long after glow. The structural studies carried out using XRD technique shows that the sample is single phased in nature and it gets crystallized into monoclinic phase with standard JCPDS 34-0379 card. The oxide formation was examined using FTIR technique. UV–Visible spectroscopy has been used to study the optical band gap of material, it’s value in the current case, Sr_1−x_Al_2_O_4_:Eu_x_ (x = 0.05) is 3.78 eV. Scanning electron microscopy (SEM) and transmission electron microscopy (TEM), confirm the formation of nano particle, with average particle size around 6–25 nm. The elemental composition was confirmed by using Energy Dispersive X-ray (EDX) technique. The photo-luminescence study revealed that it gives broad emission spectra using excitation wavelength λ_ex_ = 365 nm. It is observed that the Sr_1−x_Al_2_O_4_:Eu_x_ (x = 0.05) phosphor give maximum emission intensity and it can be regulated as green (0.23, 0.49) emission with the colour temperature 3224 K, CRI 78, and colour purity 60.69%. The spectra are intense and lie in the visible range. The green lights can regulate the circadian rhythm through melatonin, and it is also suitable for green LED and other optoelectronic devices. The Sr_1−x_Al_2_O_4_:Eu_x_ (x = 0.00 and 0.05) phosphor behaves like eco-materials, because nano particles of Sr_1−x_Al_2_O_4_:Eu_x_ (x = 0.05) does not show antimicrobial activity.

## Introduction

Phosphor materials are being used more widely as a durable, cost-effective source of energy on a global basis. Light-emitting diodes (LEDs) are anticipated to reduce light energy use by 15% by 2020 and 40% by 2030, totalling 3.0 quads in 2030 alone, compared to today's estimates, according to the US Department of Energy, resulting in a $26 billion energy savings^1^. As a result, there would be a nearly 190 million metric tonne decrease in carbon dioxide (greenhouse gas) emissions. Luminescence, which has revolutionised everything from overhead fluorescent tubes to incandescent light bulbs, may become extinct in the coming years, according to the United States Department of Energy^2^. Because of their inherent qualities, such as low energy consumption, long life, high chemical stability, and environmental friendliness, phosphors converted white light-emitting diodes and other light-emitting diodes are outstanding materials for solid lighting technology and could be used in future lighting devices^3^. Due to their outstanding luminous properties, rare earth and alkaline earth aluminates phosphorescent materials have a wide range of applications in solid-state lighting, display components, and other fields. Alkaline earth aluminates make the perfect chemical host material for enhancing luminescence in today's luminescence-based technologies. The host lattice's structure affects the luminescence, which can happen anywhere in the electromagnetic spectrum from ultraviolet to infrared. The long-lived luminescence property is demonstrated by the photoluminescence of rare-earth-doped alkaline earth aluminates, MAl_2_O_4_ (M = Ca, Sr, Ba)^4^. The researchers have gained interest in long afterglow characteristic of rare earth-doped alkaline earth aluminates phosphors. Luminescent paint, emergency device safety indicators, electronic instrument dial pads, vehicle dials and panels, writing and printing inks, plasma display phosphors, and many other products use phosphor ingredients^5^. SrAl_2_O_4_ is commonly produced using a solid state reaction method that necessitates annealing at high temperatures, usually above 1000 °C, for extended periods of time, ranging from several hours to days, in order to form crystals and produce particle sintering^6–8^. The literature has already reported on the sol–gel method, crystallisation of spray-dried amorphous precursors precipitation, flame spray pyrolysis, and other synthesis techniques^9–12^. The combustion method, which involves an exothermic reaction between metal nitrates and an organic fuel, is employed in this study since it is a rapid and energy-efficient process. The combustion process self-replicates once the raw material solution is heated to a low temperature and ignited to produce the required chemical^4,13^. The discussion serves as the inspiration for the nano-scale phosphor preparation. In the present investigation the Sr_1−x_Al_2_O_4_:Eu_x_ (x = 0.00, 0.01, 0.03, 0.05, 0.07, and 0.09) phosphor nanocrystalline powder were synthesized by using urea fuel combustion method. The structural, optical, morphological, and photoluminescence studies of the phosphor sample were investigated and the antibacterial property has been investigated for the first time for Sr_1−x_Al_2_O_4_:Eu_x_ (x = 0.00 and 0.05) phosphors. Our study puts forward an eco-friendly material which is suitable to be used as an optical coating agent for making solid state luminescent devices^14,15^.

## Experimental

### Sample preparation

To synthesize Sr_1−x_Al_2_O_4_:Eu_x_ (x = 0.00, 0.01, 0.03, 0.05, 0.07, and 0.09) phosphor, urea fuel combustion was employed. In the synthesis process, nitrates such as strontium nitrate [Sr(NO_3_)_2_], aluminium nitrate [Al(NO_3_).H_2_O], oxides such as europium oxide [Eu_2_O_3_], and urea [CO(NH_2_)_2_] were utilized as starting materials. The Europium oxides were converted to nitrates by dissolving them in 5 ml of concentrated HNO_3_.

1 $${\text{Eu}}_{2} {\text{O}}_{3} + 6{\text{HNO}}_{3} \to 2{\text{Eu}}({\text{NO}}_{3} )_{3} + 3{\text{H}}_{2} {\text{O}}$$

Urea was combined with the initial components in stoichiometric ratios, and the mixture was then mashed with a mortar-pestle to create a paste. Using a vertical furnace, the paste was delivered to the crucible and burned at 580 °C^16^. The solution catches fire and turns into white foam (ash) in a few seconds. The entire reaction takes 5–10 min to complete. Using a mortar and pestle, the ashes left behind from burning the solution were turned into a fine nanopowder^17^.

**Figure 1:**
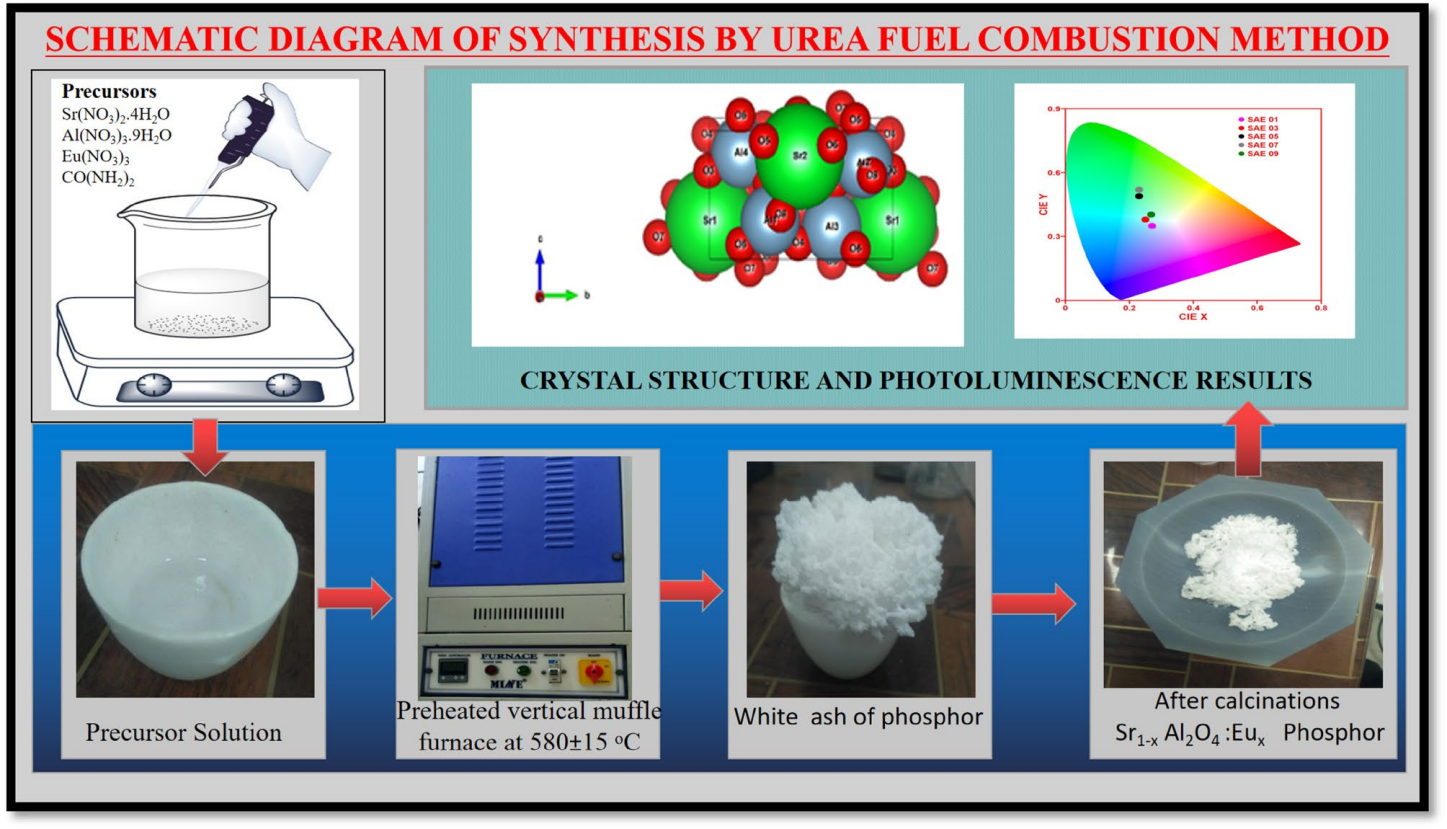
Schematic diagram for urea fuel Combustion synthesis method.

The chemical reaction of these reactants is shown in Eq. (2) which is for the Sr_1−x_Al_2_O_4_:Eu_x_ (SAE) phosphor synthesized with the urea fuel combustion method as shown in Fig. 1.

2 $$\left( {1 - x} \right)Sr(NO_{3} )_{2} \cdot 4H_{2} O + \left( x \right)Eu(NO_{3} )_{3 } + Al(NO_{3} )_{3} \cdot 9H_{2} O \to Sr_{1 - x} Al_{2} O_{4} :Eu_{x} + \left( {H_{2} + N_{2} + O_{2} + NH_{3} + H_{2} O} \right) \uparrow$$

The samples synthesized with different molar ratios of Eu^2+^ ions such SrAl_2_O_4_:Eu_0.00_, Sr_0.99_Al_2_O_4_:Eu_0.01_, Sr_0.97_Al_2_O_4_:Eu_0.03_, Sr_0.95_Al_2_O_4_:Eu_0.05_, Sr_0.93_Al_2_O_4_:Eu_0.07_ and Sr_0.91_Al_2_O_4_:Eu_0.09_ by urea fuel combustion method and for simplicity the samples coded as SAE 0.00, SAE 0.01, SAE 0.03, SAE 0.05, SAE 0.07 and SAE 0.09 respectively.

### Characterization techniques

The crystalline phase purity of Sr_1−x_Al_2_O_4_:Eu_x_ (x = 0.00, 0.01, 0.03, 0.05, 0.07, and 0.09) phosphors were characterized by X-ray diffraction pattern; it was obtained using Bruker D8 Advance X-ray diffractometer with CuKα (1.5406 Å) radiation. The diffraction pattern was recorded between 10° and 70° (2θ) by using step size at 0.02680 and Estimated Scan Time of 3832 s/step during the XRD measurement. The structural parameters were estimated by using Full-prof program. The surface morphology of samples was studied using a scanning electron microscope (SEM, NOVA NANOSEM 450). For the purpose of confirming the elemental composition, an energy dispersive X-ray (EDX) spectrometer (Oxford-EDX system INCA 250 EDS linked with SEM, NOVASEM 450) was employed. Images of transmission electron micrographs (TEM) were obtained by utilizing a TEM-TECNAI G2 T30 (S-TWIN) electron microscope with a voltage range of 100–400 kV, which was employed for measuring the particle crystal picture, HRTEM image, selected area electron diffraction (SAED) image, crystal structure, voids, pores, particle size defect, and other properties of Sr_1−x_Al_2_O_4_:Eu_x_ (x = 0.05) phosphor. Fourier transform infrared spectroscopy was used to examine the chemical bonding behavior of materials (FTIR-6300 spectrometer equipped with NRS-3300 laser, Jasco, Japan). The optical characterization was done by using UV–VISIBLE spectrophotometer (Labindia Analytical UV3092) and the photoluminescence (PL) was recorded on spectrofluorophotometer (Horiba scientific Instrument Fluoromax-4 spectrofluorometer) with a spectral slit width of 1.0 nm using a 450 W xenon arc lamp as an exciting source.

### Antibacterial studies

In this investigation, gram-negative bacteria *Escherichia coli* (*E. coli*) and gram-positive bacteria *Staphylococcus aureus* (*S. aureus*) were employed as pathogenic bacterial strains. The antibacterial activity of Sr_1−x_Al_2_O_4_:Eu_x_ (x = 0.00 and 0.05) phosphors were evaluated using the agar well diffusion method. In sterilized Petri dishes, nutrient media was added. The each bacterial strain was disseminated separately on the agar medium and seeded into Luria–Bertani (LB) broth along with the respective clinical isolates for 24 h-incubated cultures. In each Petri plate, wells with a 6 mm diameter are created using a sterile cork borer and under aseptic circumstances. Nanoparticle concentrations of 1 mg/ml were utilized to measure the activity of the particles. Using sterile micropipettes, the nanoparticles are mixed with distilled water and added to the wells. The common antibiotic ciprofloxacin (used as a positive control) was tested against the pathogens simultaneously, after which the plates were incubated for 24 h at 37 °C. Each well's zone of inhibition was examined after the incubation period, and the results were recorded.

## Results and discussion

### X-ray analysis

**Figure 2:**
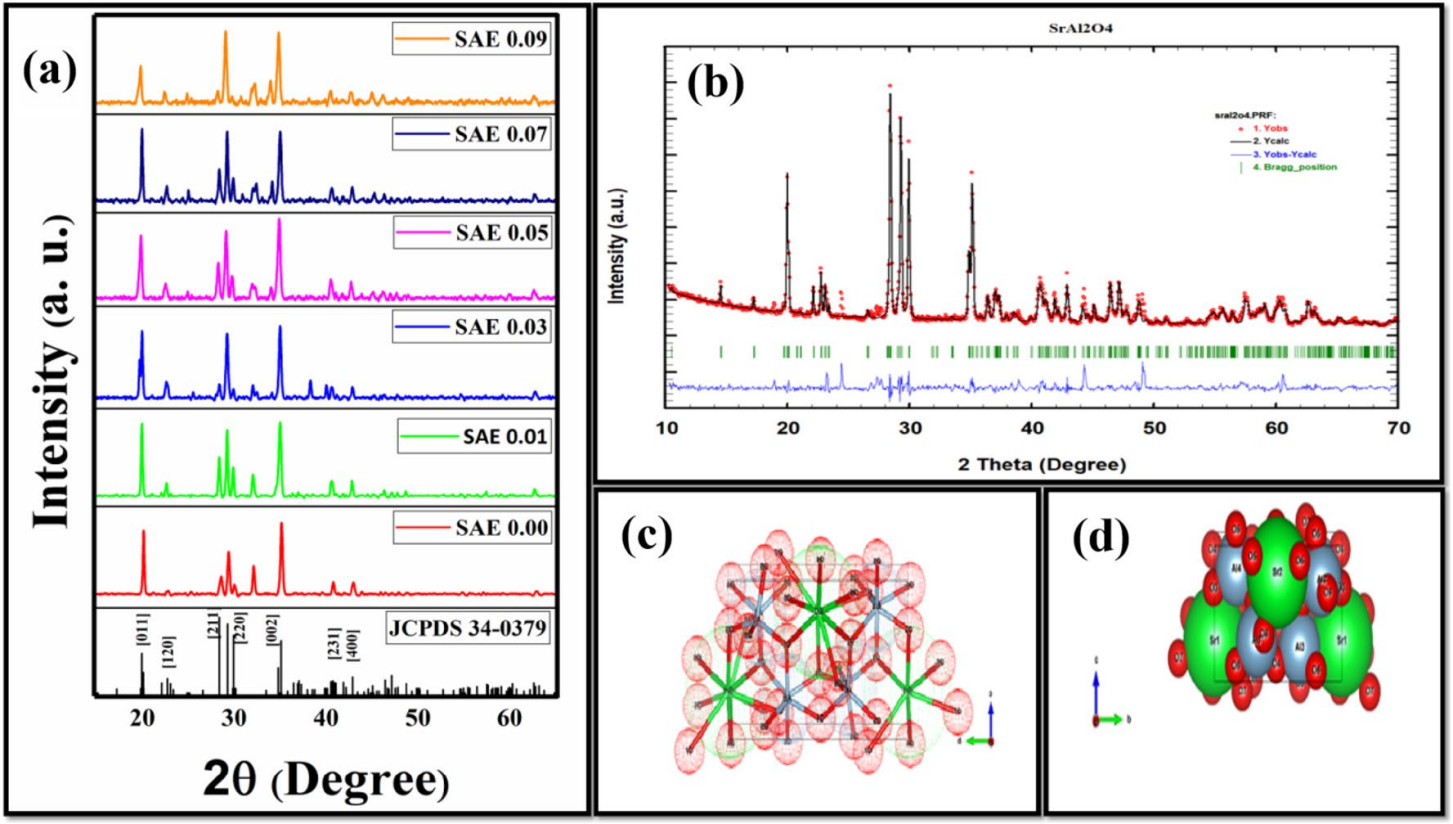
XRD spectra (**a**) Typical Rietveld refinement Crystallographic image of SrAl_2_O_4_ (**b**) Crystal structure of SrAl_2_O_4_ (**c**,**d**).

The synthesized Sr_1−x_Al_2_O_4_:Eu_x_ (x = 0.00, 0.01, 0.03, 0.05, 0.07, and 0.09) phosphor’s (SAE phosphor) dominating peaks are completely matches that of the reference JCPDS Card 34-0379. The single-phase character of the synthesized sample was shown by the XRD data obtained in the angle range of 10° to 70° displayed in Fig. 2a. Additionally, it was noted that the sample had a monoclinic structure with the space group P 21/n. The sample is crystalline in nature according to the characterization peak's intensity; however the sample's broad peaks show lesser average crystalline size.

### Rietveld refinement analysis

The Rietveld refinement was used to obtain the crystal structure of the synthesised phosphors with the help of the Full-Prof programme. We refined the SrAl_2_O_4_ sample using the pseudo Voigt function, as displayed in Fig. 2b. The synthesised sample's refining results and the measured and computed values match. The refinement supported the existence of the pure monoclinic structure with the P 21/n space group indicated in Fig. 2c,d. The calculated refined phase parameters are as follows: a = 8.4423 Å, b = 8.8243 Å, c = 5.1544 Å, α = 90.00°, β = 93.36°, γ = 90.00°, V = 383.3143 Å^3^ and χ^2^ = 4.8. For sample SrAl_2_O_4_ (SAE 0.00), the goodness of fit (GOF) value was determined to be 2.8. All these data are analogues to the values reported by the authors in different works of literature^18,19^. The refinement parameters and their values are listed in Table 1. A comparison of refinement crystallographic data for atom parameters of SrAl_2_O_4_ phosphor is listed in Table 2.

**Table 1:** Typical Rietveld refinement crystallographic data of SrAl_2_O_4_.

S. no.	Refinement parameters	Refinement parameter values
1	Empirical formula	SrAl_2_O_4_
2	Formula weight	205 g/mol
3	Crystal system	Monoclinic
4	Space group	P 21/n
5	Unit cell parameters	a = 8.4423 Å b = 8.8243 Å c = 5.1544 Å, α = 90.000°, β = 93.367°, γ = 90.000°
6	Vol	383.3143 Å^3^
7	Z	383.3143 Å^3^
8	Calculated density	3.310 g/cm^3^
9	R_p_	20.4
10	R_wp_	22.5
11	R_exp_	8.15
12	Chi (χ^2^)	4.8
13	R_Bragg_-factor	9.42
14	D_W-Stat_.	0.6272
15	D_W-exp_	1.8319
16	N-sigma of the GoF	132.592
17	Goodness of fit	2.8

**Table 2:** Comparison of refinement crystallographic data for atom parameters of SrAl_2_O_4_ phosphor.

Atoms	Valence state	Atomic co-ordinate			B_iso_	Occupancy/sof	Mult
		x	y	z			
Sr1	2+	0.49891	0.00415	0.25702 (0)	3.26599 (0)	1.19373	2
Sr2	2+	0.02642	0.98131	0.20711	1.08341	0.97732	2
Al1	3+	0.17849	0.82556	0.70000	15.12612	1.15724	2
Al2	3+	0.81046	0.81875	0.72882	2.28465	1.53862	2
Al3	3+	0.73563	0.64105	0.23710	-0.23700	1.16699	2
O1	2+	0.24081	0.17966	0.46454	11.72810	0.74725	2
O2	2+	0.69660	0.34847	0.60899	7.79535	1.05501	2
O3	2+	0.32209	0.52419	0.34366	4.81708	0.79570	2

These formulas are used to compute the crystallite sizes (D), Dislocation density (δ), Micro strain (ε) and inter-planner spacing (d). The values of crystallite sizes (D), Dislocation density (δ), Microstrain (ε) and inter-planner spacing (d) for Sr_1−x_Al_2_O_4_:Eu_x_ (x = 0.00, 0.01, 0.03, 0.05, 0.07, and 0.09) phosphor (SAE phosphor) are given in the Table 3^17,20–22^.

3 $$Crystallite Size D = \frac{0.9 \uplambda }{{\upbeta\,{\text{COS}}\,\uptheta }}$$

4 $$Dislocation Density \delta = \frac{n}{{D^{2} }}$$

5 $$Microstrain \epsilon = \frac{\beta }{{4 {\text{Tan}} \theta }}$$

6 $$Interplanner Spacing d = \frac{n\lambda }{{2 {\text{Sin}} \theta }}$$

where D is the crystallite size, n equates to unity, resulting in the lowest possible dislocation density, λ is the X-beam wavelength, θ is the Bragg's diffraction angle, and β is the full width at half maxima (FWHM), expressed in radians^23^.

**Table 3:** Structural parameters—crystallite size D (nm), Dislocation density, Microstrain and Inter-planner spacing d (nm) of Eu^2+^ doped Sr_1−x_Al_2_O_4_:Eu_x_ (x = 0.00, 0.01, 0.03, 0.05, 0.07, and 0.09) phosphor (SAE phosphor).

S. no.	Sample code	Crystallite size	Dislocation density	Microstrain	d Spacing
		D (nm)	δ × 10^–3^ (nm^-2^)	ε × 10^–3^	(nm)
1	SAE 0.00	34.59	0.96	3.09	0.27
2	SAE 0.01	34.34	0.90	3.95	0.30
3	SAE 0.03	30.14	2.21	4.89	0.27
4	SAE 0.05	26.95	1.54	4.46	0.26
5	SAE 0.07	30.55	1.28	4.57	0.30
6	SAE 0.09	32.61	1.24	4.32	0.28

**Figure 3:**
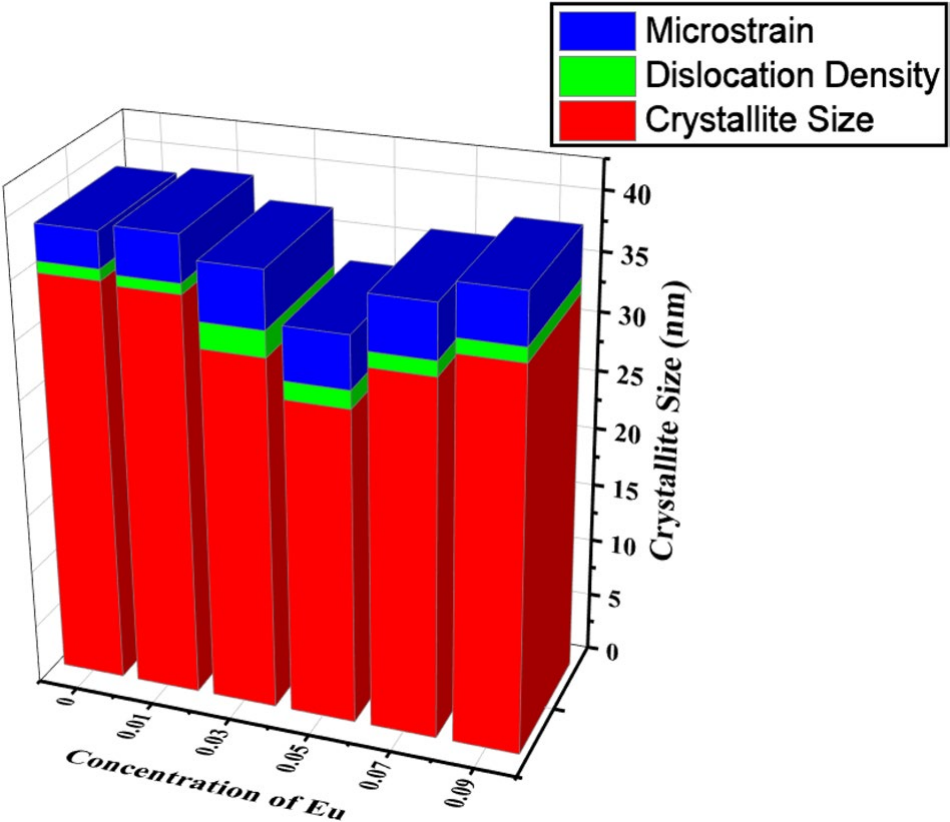
Variation of Microstrain, Dislocation Density and Crystallite Size with Concentration of Eu for Sr_1−x_Al_2_O_4_:Eu_x_ (x = 0.00, 0.01, 0.03, 0.05, 0.07, and 0.09) phosphor (SAE phosphor).

From the Table 3 it is shown in Fig. 3 that when we start increasing the concentration of europium in our host SrAl_2_O_4_ (SAE 0.01 to SAE 0.05) it’s average crystallite size is decreasing. This is due to their variable ionic radii; the change of Eu particles with the Sr site is lower. However, there is a slight difference in their ionic radii Sr^2+^ (1.13 Å) and Eu^2+^ (1.06 Å) substituting for the smaller Al^3+^ (0.53 Å) ions in the host lattice. The Eu^2+^ ion is incorporated into SrAl_2_O_4_ lattice rather than interstitial sites^24,25^. The average crystallite size is minimum for SrAl_2_O_4_:0.05Eu (SAE 0.05), due to this the luminescence property also changes abruptly Fig. 11b.

### SEM analysis

**Figure 4:**
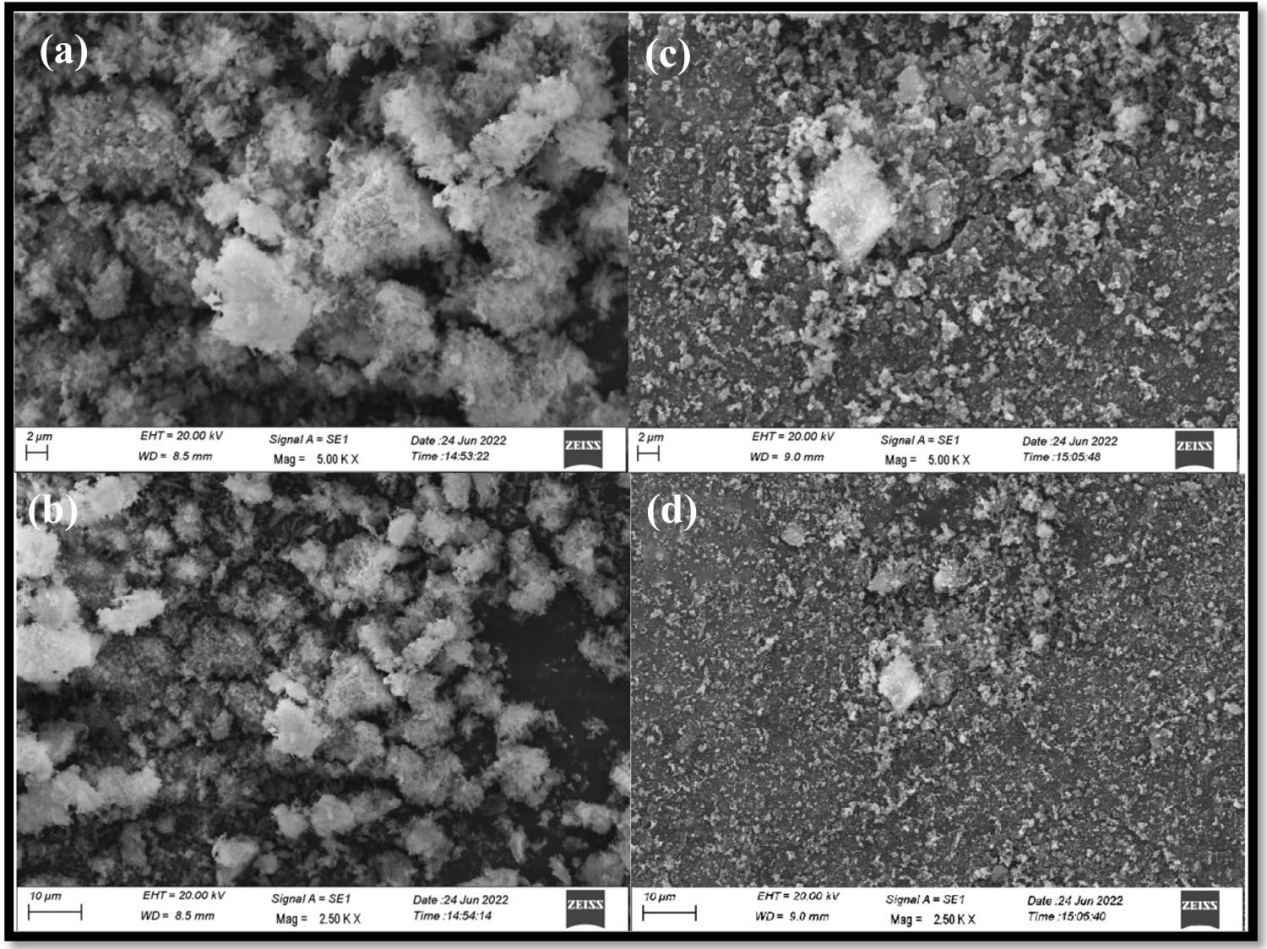
(**a**,**b**) Pure SrAl_2_O_4_ (SAE 0.00) phosphor, **(c**,**d) **Eu^2+^ doped Sr_1−x_Al_2_O_4_:Eu_x_ (x = 0.05) (SAE 0.05) phosphor SEM image.

The SEM images of the Sr_1−x_Al_2_O_4_:Eu_x_ (x = 0.00 and 0.05) phosphor (SAE0.00 and SAE 0.05 phosphor) are displayed in Fig. 4a,b and c,d, respectively. The sample's microstructure exhibits characteristics of combustion as it occurs naturally. Pores are created with the production of microscopic particles close to the pores while a gas is exiting under high pressure during combustion. Figure 4a–d illustrates the uneven and non-uniform forms of the particles.

**Figure 5:**
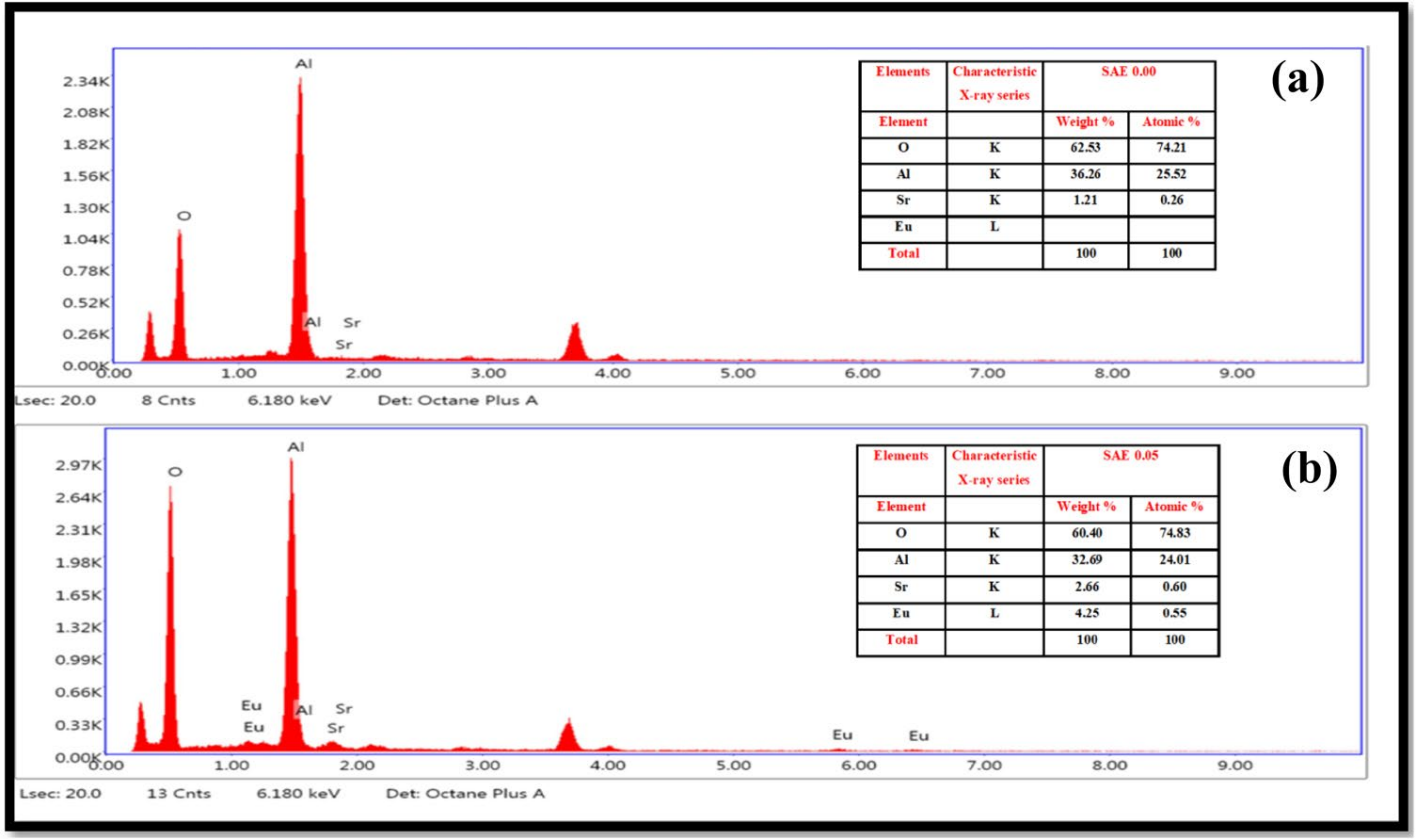
(**a**) Typical EDX spectra Sr_1−x_Al_2_O_4_:Eu_x_ (x = 0.00) (SAE 0.00) and (**b**) Sr_1−x_Al_2_O_4_:Eu_x_ (x = 0.05) phosphor (SAE 0.05) material.

The elemental composition and distribution of the sample can be determined from the Energy Dispersive X-ray (EDX) spectra. The EDX spectra of the SAE 0.00 and SAE 0.05 phosphor (Sr_1−x_Al_2_O_4_:Eu_x_ (x = 0.00, 0.05) phosphor) samples are depicted in Fig. 5a,b, respectively. The EDX spectra show that oxygen (O), strontium (Sr), aluminium (Al), europium (Eu), and these metals are the phosphor’s main constituents. Quantitative EDX analysis reveals that while the percentage contribution of the other elements remained constant, the concentration of Sr decreased as a result of the rise in dopant Eu^2+^. This agrees well with the theoretically calculated stoichiometry. The EDX spectra's prominent peaks serve as proof that the elements are present in the synthesized phosphor.

**Figure 6:**
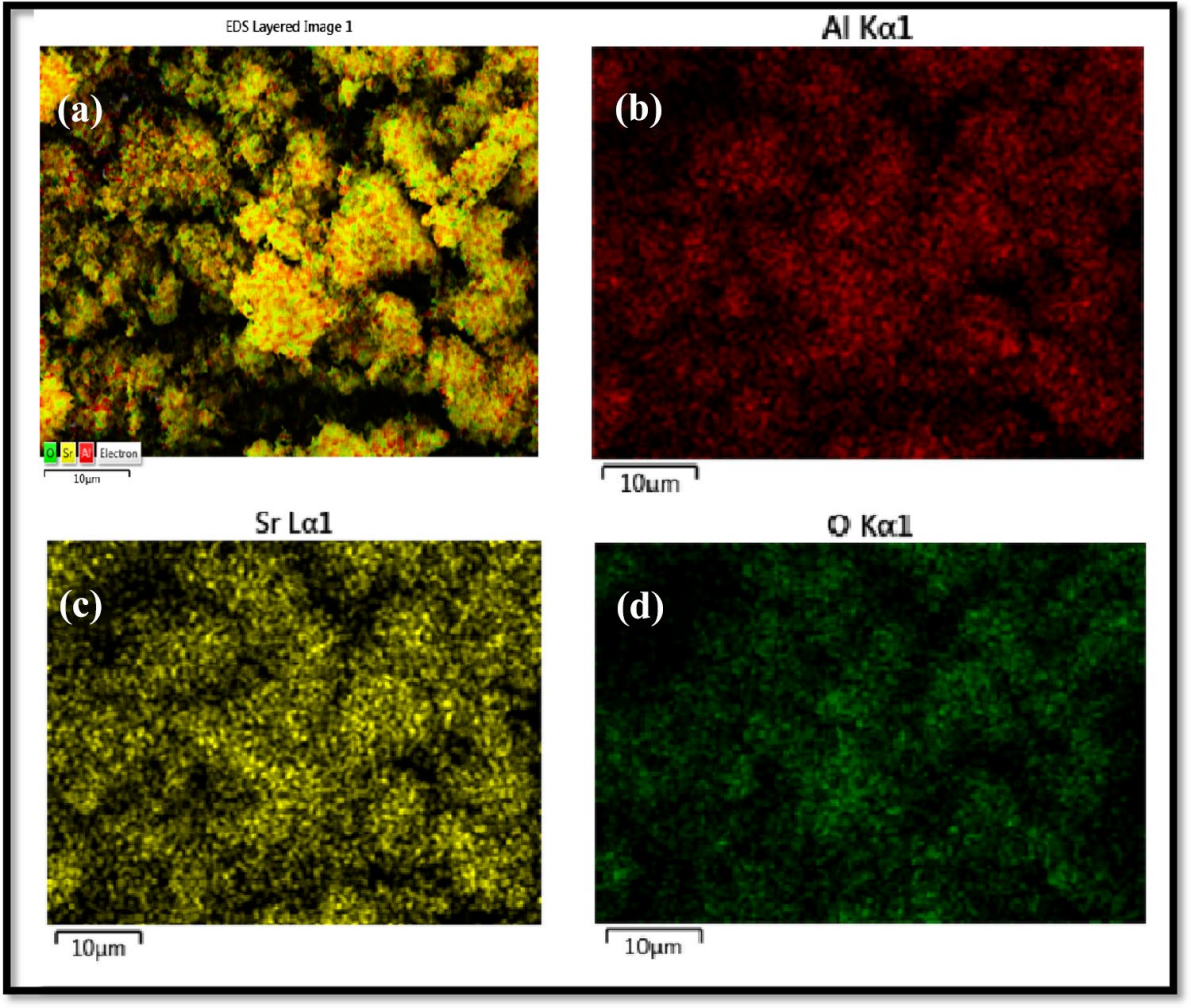
(**a**–**d**) Typical elemental mapping images forpure Sr_1−x_Al_2_O_4_:Eu_x_ (x = 0.00) phosphor (SAE 0.00).

**Figure 7:**
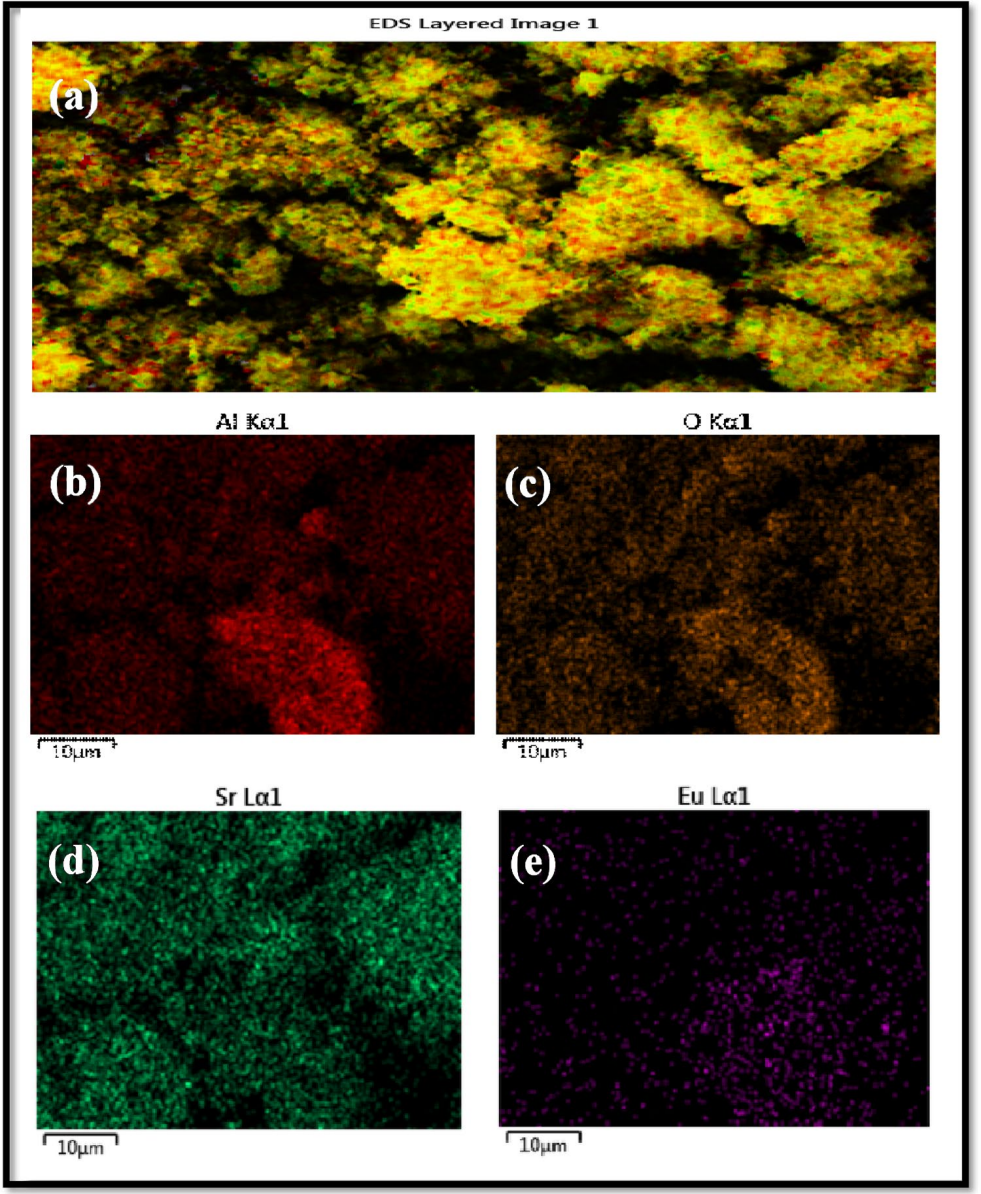
(**a**–**e**) Typical elemental mapping images for pure Sr_1−x_Al_2_O_4_:Eu_x_ (x = 0.05) phosphor (SAE 0.05).

The typical elemental mapping images of Sr_1−x_Al_2_O_4_:Eu_x_ (x = 0.00, 0.05) phosphor (SAE 0.00 and SAE 0.05 phosphor) samples material are shown in Figs. 6a–d and 7a–e respectively. The elemental mappings show the homogeneous distribution of nano particles in crystal structure of synthesized phosphor.

### TEM analysis

**Figure 8:**
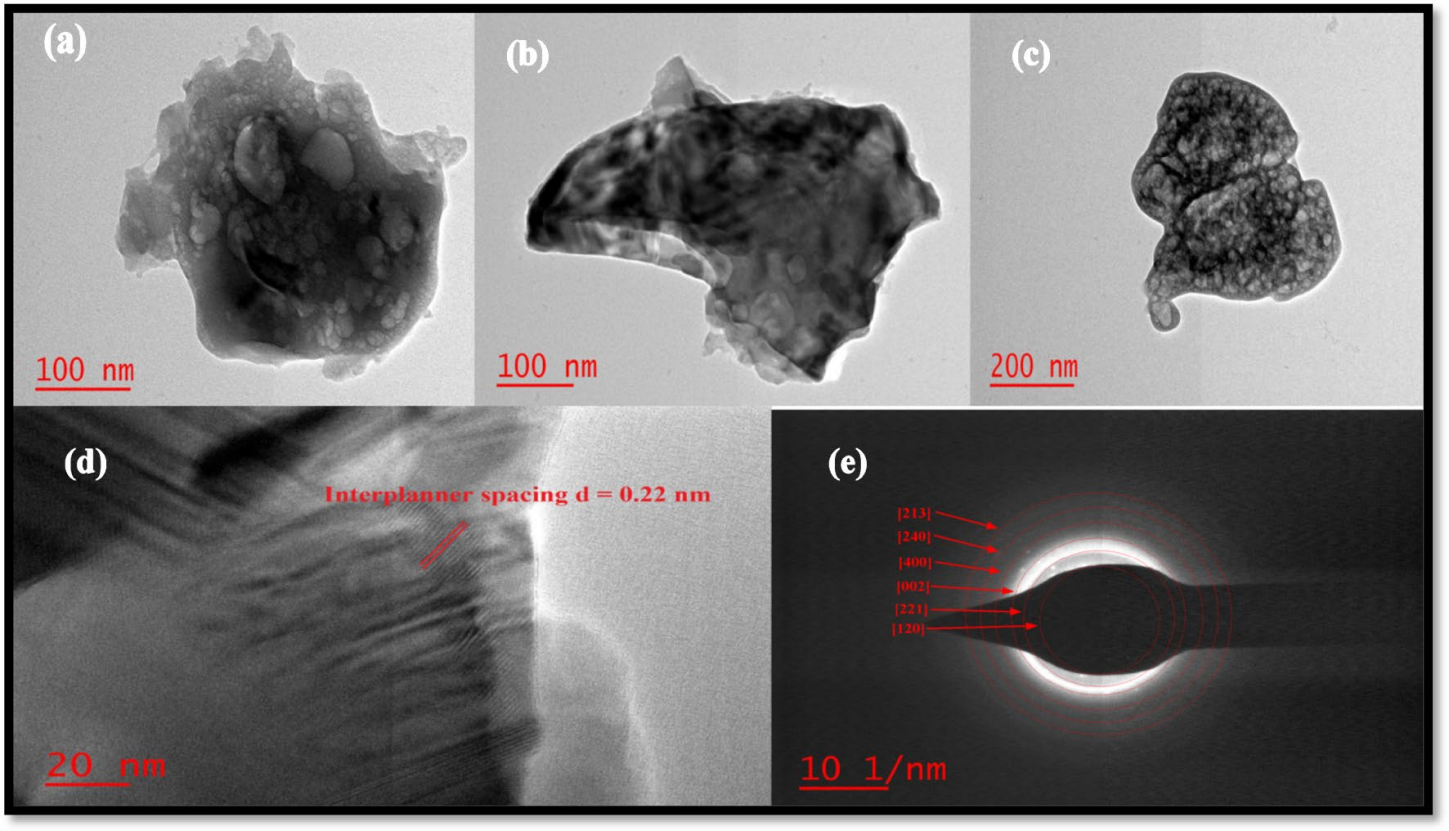
Sr_1−x_Al_2_O_4_:Eu_x_ (x = 0.05) phosphor TEM image (**a**–**c**) and HRTEM image (**d**) and SAED pattern (**e**).

Transmission electron microscopy was used to measure the particle crystal imaging, HR image, and selected area electron diffraction (SAED) image. The specifics of defects in crystal structure, voids, pores, and particle size, etc. The TEM micrograph shows the growth of irregular shape of agglomerated nanoparticles as shown in Fig. 8a–c. The average particle size is found around 6–25 nm (Calculated by Image J software). The associated SAED pattern (Fig. 8e) makes it obvious that the diffraction pattern is from a nano phosphor assembly since it displays distinct rings. Table 4 displays the inter-planner spacing determined from the TEM SAED pattern. Using the Image J software, the fringe width of the HRTEM image was estimated. Figure 8d displays a typical HRTEM image of a phosphor sample. The homogeneous lattice fringe with estimated inter-planner spacing d = 0.22 nm by using TEM data and observed inter-planner spacing d = 0.222 nm for (231) plan from XRD result is highly matched (Supplementary Table) for Sr_1−x_Al_2_O_4_:Eu_x_ (x = 0.05) phosphor (SAE 0.05)^26^.

**Table 4:** Inter-planner spacing (d-spacing) for Sr_1−x_Al_2_O_4_:Eu_x_ (x = 0.05) phosphor (SAE 0.05 phosphor) from TEM SAED pattern.

	1/D or 1/2r	1/r	r	d spacing	(h kl)
	nm^−1^	nm^−1^	nm	nm	
1	13.22	26.45	0.037	0.378	(120)
2	16.77	33.55	0.029	0.298	(221)
3	19.30	38.60	0.025	0.259	(002)
4	23.07	46.14	0.021	0.216	(400)
5	26.57	53.15	0.018	0.188	(240)
6	30.02	60.05	0.016	0.166	(213)

### FTIR analysis

**Figure 9:**
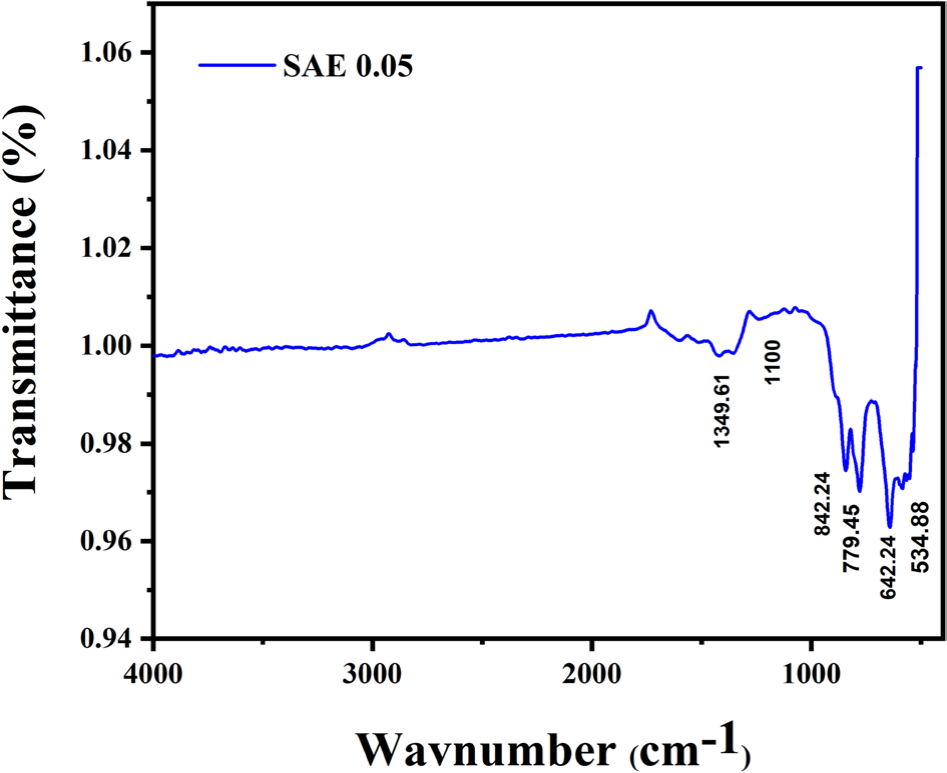
FTIR spectrum of Sr_1−x_Al_2_O_4_:Eu_x_ (x = 0.05) phosphor (SAE phosphor).

The Fourier transform Infra-red spectra (FTIR) for Sr_1−x_Al_2_O_4_:Eu_x_ (x = 0.05) phosphor (SAE 0.05 phosphor) is shown in Fig. 9. The Fourier transform Infra-red spectra were recorded in the wave number range of 400–4000 cm^−1^. The monoclinic crystal structure of SrAl_2_O_4_ was determined by a sequence of absorption peaks in the 400–900 cm^−1^ range. The Al=O, Sr=O, and Sr–O–Al bond vibrations are responsible for the bands between 350 and 1000 cm^−1^, which are all connected to the infrared active vibration modes of SrAl_2_O_4_ phosphor. The band at 534 cm^−1^ is produced by the symmetric bond of O–Al–O, but the anti-symmetric stretching bands between 588 and 845 cm^−1^ are produced by the vibrations of Sr–O, leading to the conclusion that the band at 842.24 cm^−1^ is most likely Sr–O. The inherent active IR vibration modes of strontium aluminates are commonly seen in the bands below 1000 cm^−1^. The anti-symmetric stretching bands of Sr–O at 642.24 cm^−1^, 779.45 cm^−1^, and 842.24 cm^−1^ are absorption bands^27^.

### UV–VISIBLE analysis

The Sr_1−x_Al_2_O_4_:Eu_x_ (x = 0.05) was examined using a UV–VISIBLE spectrophotometer to determine its optical bandgap. The diffuse reflectance spectroscopy (DRS) is a straight forward and effective spectroscopic tool for this purpose. Diffuse reflectance spectroscopy was used to examine the UV–visible absorption spectra of the Sr_1−x_Al_2_O_4_:Eu_x_ (x = 0.05) phosphor that has been doped with Eu^2+^. The Kubelka–Munk function was used to estimate the band gap. In the parabolic band structure, the E_g_ and absorption coefficient are related via the Tauc relation. The statement provides the Tauc relation for a material with a direct band gap, the Tauc relation is given by the statement^28^.

7 $$\upalpha {\text{h}}\upnu = {\text{A}} ({\text{h}}\upnu - E_{g} )^{{\text{n}}}$$

The linear absorption coefficient is α, the light frequency is υ, and the proportionality constant is A. The power of the parenthesis, n, is taken to be 1/2 for direct band gap materials. The perfect diffuse scattering of incoming light causes the absorption coefficient K to equal 2. In this illustration, assuming the scattering coefficient S is constant with respect to wavelength, the Kubelka Munk function is proportional to the absorption coefficient. Using the above equation, we get the following relationship^29^:

8 $$[{\text{F}}({\text{R}}_{\infty } ){\text{h}}\upnu ]^{2} = {\text{A }}({\text{h}}\upnu - E_{g} )$$

**Figure 10:**
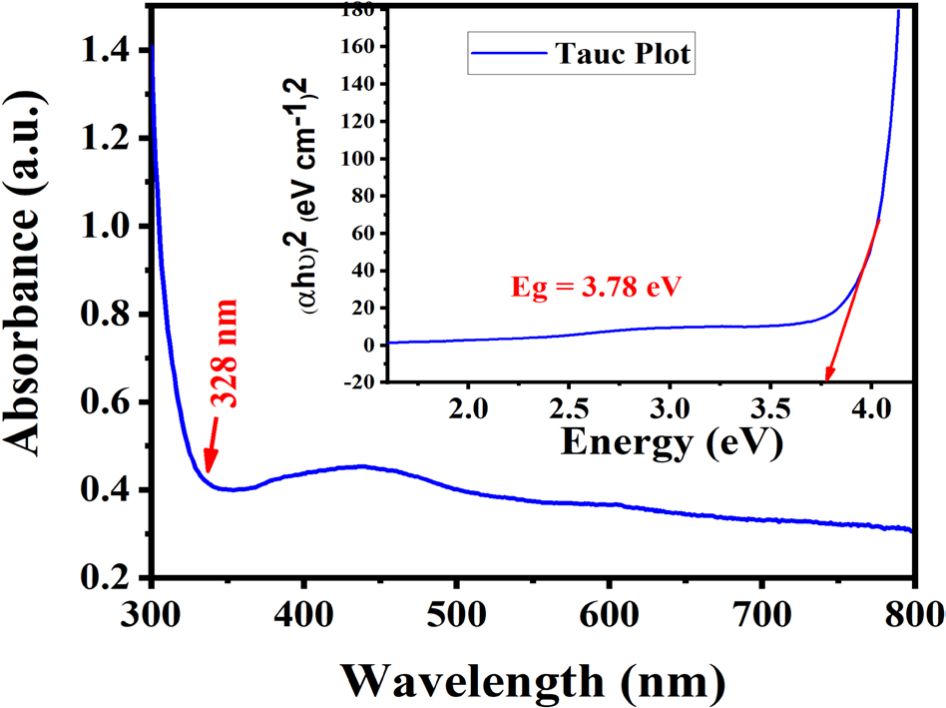
UV–Visible spectrum and Tauc Plot for Sr_1−x_Al_2_O_4_:Eu_x_ (x = 0.05) phosphor (SAE phosphor).

As demonstrated in Fig. 10, the energy-band gap of the powder sample is easily determined using the (αhυ)^2^ vs. hυ graph. The energy axis is intersected by an extrapolated straight line along the sharp edge of the curve and the optical band gap in this case (SAE 0.05) is 3.78 eV. Because of the doping of the Eu ion in the host crystal lattice, the band gap is smaller than the band gap of host material reported in literature^30^.

### Photoluminescence (PL) analysis

**Figure 11:**
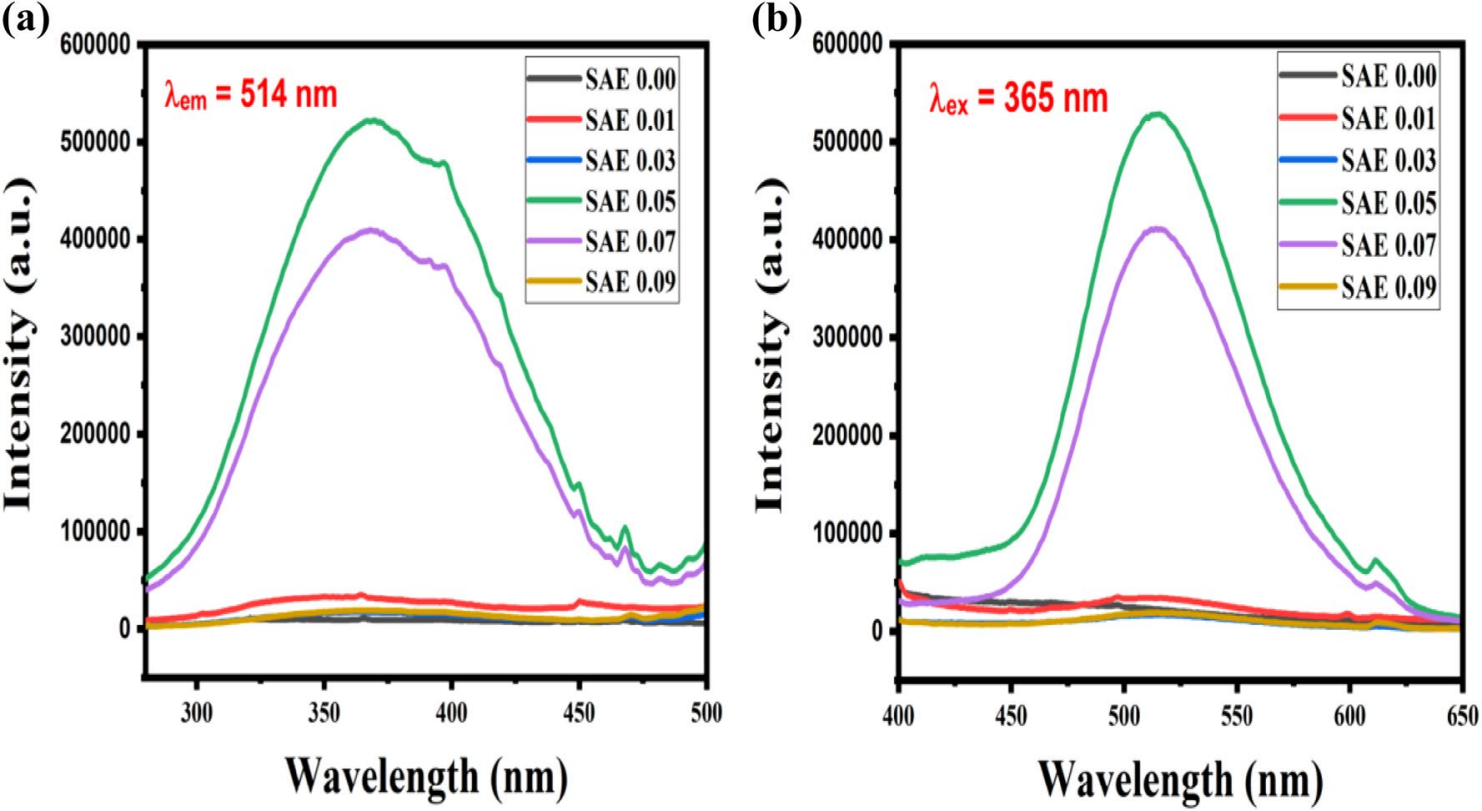
(**a**) PL excitation and (**b**) emission spectra of Sr_1−x_Al_2_O_4_:Eu_x_ (x = 0.00, 0.01, 0.03, 0.05, 0.07, and 0.09) phosphor (SAE phosphor).

The excitation spectrum of Sr_1−x_Al_2_O_4_:Eu_x_ (x = 0.00, 0.01, 0.03, 0.05, 0.07, and 0.09) phosphor (SAE phosphor) synthesized via urea fuel combustion method is shown in Fig. 11a.

The emission spectra of a Sr_1−x_Al_2_O_4_:Eu_x_ (x = 0.00, 0.01, 0.03, 0.05, 0.07, and 0.09) phosphor (SAE phosphor) was captured is shown in Fig. 11b. The excitation wavelength 365 nm is used. It consists of a broad band and the emission peak at 514 nm and a small peak at 611 nm, which is imputed to typical $4{\mathrm{f}}^{6} 5{\mathrm{d}}^{1}\to 4{\mathrm{f}}^{7}$ transition of Eu^2+^ ion^16,31,32^. Observed emission spectra reveal an obvious absorption from host SrAl_2_O_4_ in the 450-600 nm region, which can be attributed SrAl_2_O_4,_ self absorption band as well as the f-d transition of Eu^2+^. When we change the concentration of activator Eu in the host SrAl_2_O_4_ the nature of emission is change according to Fig. 11b. The Sr_1−x_Al_2_O_4_:Eu_x_ (x = 0.05) phosphor concentration give maximum emission intensity.

In the case of Sr_1−x_Al_2_O_4_:Eu_x_ (x = 0.05) (SAE 0.05 phosphor), the energy band gap from photoluminescence excitation spectra was calculated with the broad emission spectra with maximum intensity is at 365 nm, and its energy band gap is 3.39 eV, which is highly matched with optical band gap, computed using UV–VISIBLE spectroscopy, 3.78 eV (Fig. 10).

**Figure 12:**
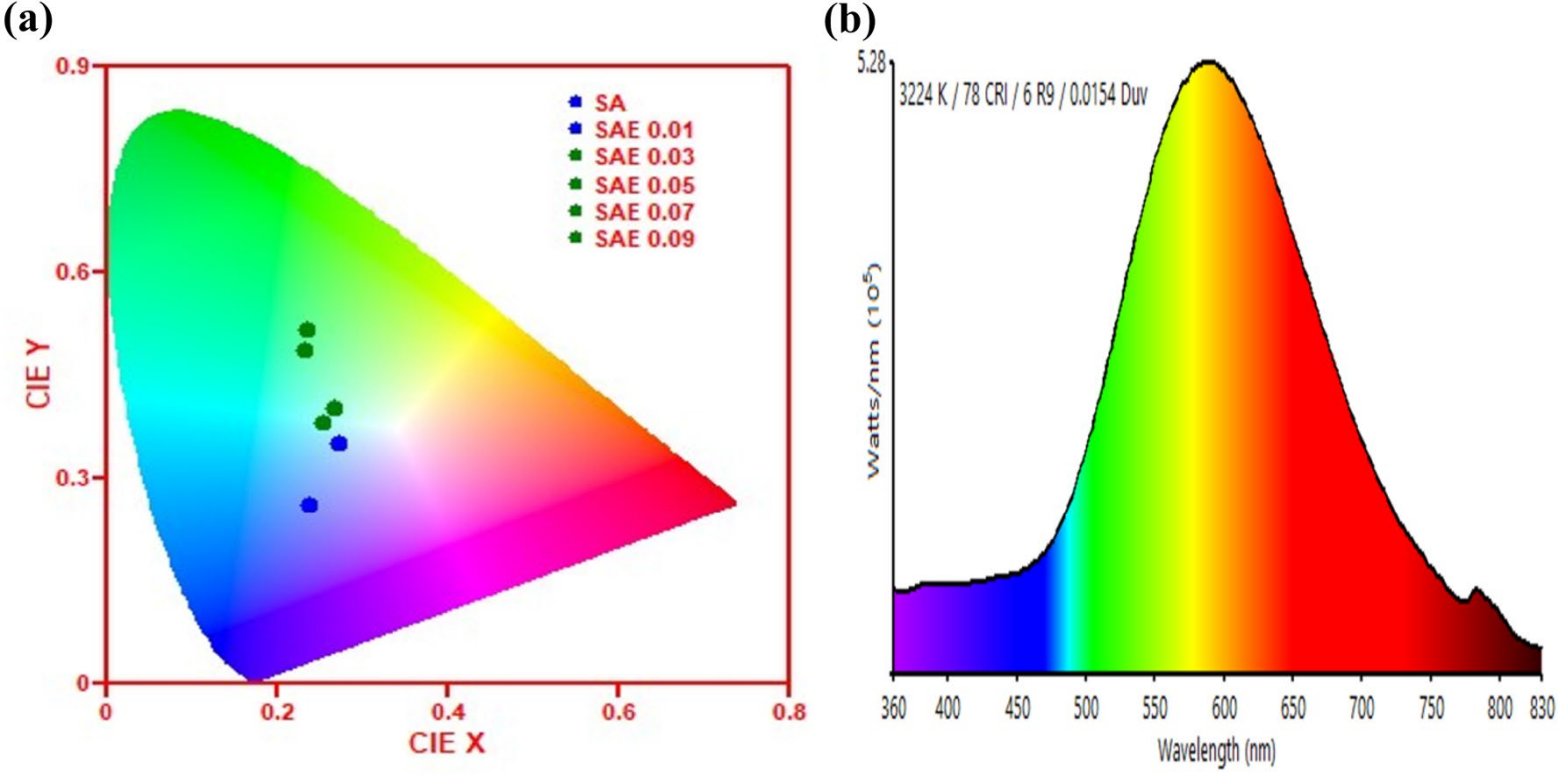
Photoluminescence CIE diagram of Sr_1−x_Al_2_O_4_:Eu_x_ (x = 0.00, 0.01, 0.03, 0.05, 0.07, and 0.09) phosphor (SAE phosphor) (**a**), Typical spectrum graph for Sr_1−x_Al_2_O_4_:Eu_x_ (x = 0.05) (SAE 0.05) phosphor (**b**).

For Visual understanding of the colour-tuneable emission of synthesized phosphors Fig. 12 show the Commission International de’ LEclarirage (CIE) diagram of Sr_1−x_Al_2_O_4_:Eu_x_ (x = 0.00, 0.01, 0.03, 0.05, 0.07, and 0.09) phosphor (SAE phosphor).

The colour purity for Sr_1−x_Al_2_O_4_:Eu_x_ have been calculated by using Eq. (9)

9 $${\text{Color}}\;{\text{purity }} = \frac{{\sqrt {\left[ {\left( {{\text{x}}_{{\text{s}}} - {\text{x}}_{{\text{i}}} } \right)^{2} + \left( {{\text{y}}_{{\text{s}}} - {\text{y}}_{{\text{i}}} } \right)^{2} } \right]} }}{{\sqrt {\left[ {\left( {{\text{x}}_{{\text{d}}} - {\text{x}}_{{\text{i}}} } \right)^{2} + \left( {{\text{y}}_{{\text{d}}} - {\text{y}}_{{\text{i}}} } \right)^{2} } \right]} }} \times 100\%$$

where (x_s_, y_s_) are the co-ordinates of phosphors reported in present work, (x_i_, y_i_) co-ordinates of white light which are (0.3101, 0.3162) and (x_d_, y_d_) are co-ordinates of dominant wavelength according to the national television standards committee (NTSC).

Each solid-state lighting product has a distinct CCT attribute that represents the colour temperature of white light. Additionally, it describes the source of the light and how warm or cold the light it emits is. The CCT value is high in the cool region and low in the warm region. However, it indicated the cool region if the CCT value of the light source was greater than 4000 K. The colour correlated temperature (CCT) is computed using McCamy empirical^33^ formula, which is given in Eq. (10)

10 $$CCT = - \;449n^{3} + 3525n^{2} + 6823n + 5520.33$$

where $n = \frac{{(x - x_{e} )}}{{y - y_{e} }}$ is the reciprocal of slope line, (x,y) are the computed CIE coordinates of Sr_1−x_Al_2_O_4_:Eu_x_and x_e_ = 0.3320, y_e_ = 0.1858 is the epicentre. The calculated CCT values of the synthesized phosphor are presented in Table 5; it is observed that the CCT values are lie between 3092–7526 K.

**Table 5:** Composition, CIE coordinate (x,y), correlated colour temperature (CCT) and colour purity for Sr_1−x_Al_2_O_4_:Eu_x_ (x = 0.00, 0.01, 0.03, 0.05, 0.07, and 0.09) phosphor (SAE phosphor).

S. no.	Composition	CIE coordinate		CCT (K)	CRI	Colour purity (%)
		x	y			
1	Sr_1−x_Al_2_O_4_:Eu_x_ (x = 0.00)	0.24	0.26	7526	96	35.34
2	Sr_1−x_Al_2_O_4_:Eu_x_ (x = 0.01)	0.27	0.35	4457	90	22.05
3	Sr_1−x_Al_2_O_4_:Eu_x_ (x = 0.03)	0.25	0.38	3927	88	32.24
4	Sr_1−x_Al_2_O_4_:Eu_x_ (x = 0.05)	0.23	0.49	3224	78	60.69
5	Sr_1−x_Al_2_O_4_:Eu_x_ (x = 0.07)	0.23	0.52	3092	76	69.00
6	Sr_1−x_Al_2_O_4_:Eu_x_ (x = 0.09)	0.27	0.40	3670	87	31.08

The importance of the light source's spectrum is described by the associated colour rendering index (CRI). It is an important parameter to define how well the spectrum of the light source is composed. An excellent quality light source has a CRI range from 75 to 100, a good quality source of light has CRI range from 56 to 75, a fair quality source of light has CRI range from 55 to 65, and a poor quality source of light has CRI range from 0 to 55 are observed^14^. In this work, we have calculated the CRI value by Eq. (11)

11 $$CRI = \frac{1}{8}\mathop \sum \limits_{i = 1}^{8} R_{i}$$

Ghosh et al. have already described the idea behind and method for calculating the colour rendering index (CRI)^34^. Calculated CRI values in this work range from 76 to 96, indicating the prepared SAE phosphor exhibits an excellent CRI range for green colour. The use of broad photo emission spectra with excellent colour index and high CCT value to obtain direct green light emitting diodes and other optoelectronic devices could be considered another possibility; the green lights can regulate the circadian rhythm through melatonin. The Table 5, lists the chromaticity coordinates, correlated colour temperature (CCT), colour rendering index (CRI) and colour purity determined from the associated emission spectrum^35,36^. It can be observed that the Sr_1−x_Al_2_O_4_:Eu_x_ (x = 0.05) phosphor can be regulated as green (0.23, 0.49) emission with the colour temperature 3224 K, CRI 78 and colour purity 60.69%.

### Antibacterial property of the phosphor

**Figure 13:**
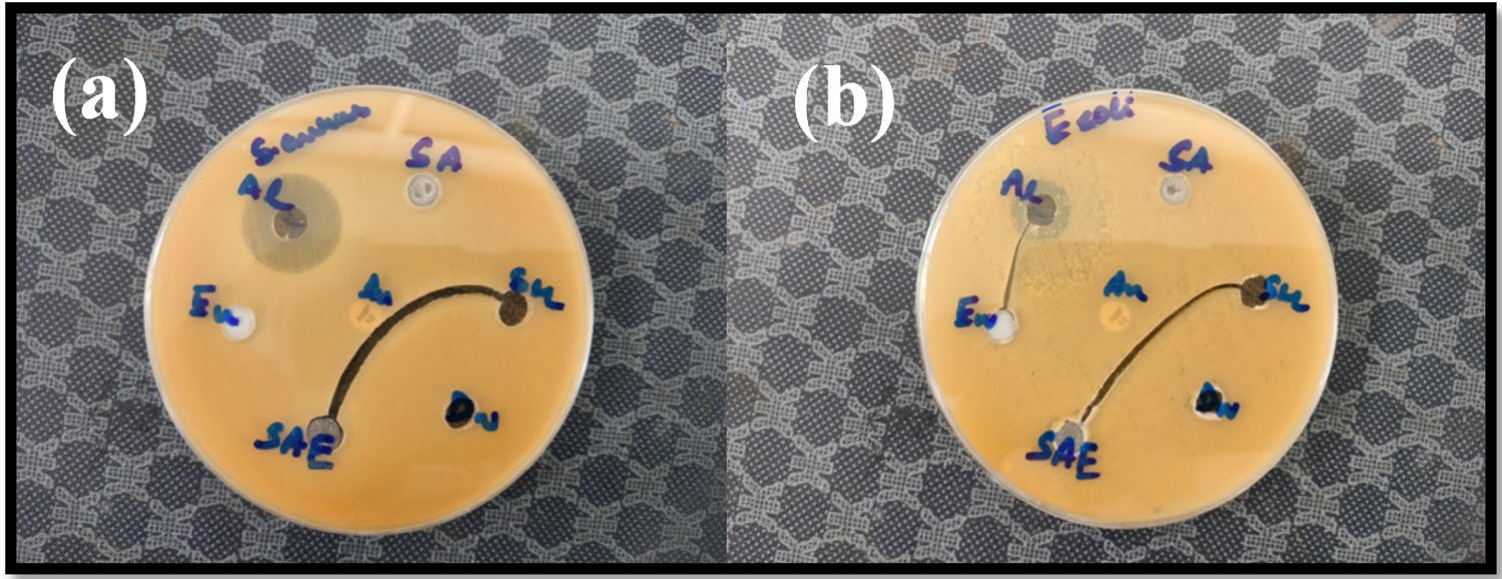
(**a**) The antibacterial activity study of SrAl_2_O_4_ (SAE 0.00) and (**b**) Sr_1−x_Al_2_O_4_:Eu_x_ (x = 0.05) phosphor (SAE 0.05 phosphor).

The antibacterial property was investigated first time for Sr_1−x_Al_2_O_4_:Eu_x_ (x = 0.00 and 0.05) phosphors. In this work the antibacterial activity of Sr_1−x_Al_2_O_4_:Eu_x_ (x = 0.00, and 0.05) phosphor (SAE 0.00 and SAE 0.05 phosphor) is observed by adopting agar well diffusion method against Gram-positive *Staphylococcus aureus* (*S. aureus*) and Gram negative *Escherichia coli* (*E. coil*) bacteria, which are tabulated in Table 6, for observing antibacterial study we have used our synthesized phosphor (SAE 0.00 and SAE 0.05 phosphor) and initial material such as Al, Sr and Eu as control. So according to an observation from the Fig. 13a,b, we have found a small inhibition zone around 5 mm for *Escherichia coli* bacteria for Al control (Fig. 13a). The Gram-positive *Staphylococcus aureus* bacteria exhibited comparative larger inhibition zone around 20 mm for Al control (Fig. 13b). The rest phosphor material and control did not show any inhibition zone for both Gram-positive *Staphylococcus aureus* and Gram-negative *Escherichia coli* bacteria. Since there are several physical parameters that can affect the antibacterial activity of the material namely particle size, morphology, shape, stability, reactivity, and chemical properties. In the last two decades, researchers, scientists, and technologists have utilised both organic and inorganic nanoparticles as antibacterial agents due to their eco-friendliness and enhanced material durability. From our study we can conclude that the Sr_1−x_Al_2_O_4_:Eu_x_ (x = 0.00, and 0.05) phosphor (SAE 0.00 and SAE 0.05 phosphor) synthesized by urea fuel combustion method is eco-friendly material, because it did not release any high toxic gas during synthesis process, therefore the synthesized material is applicable as an optical coating agent for making solid state luminescent devices^14,15^. It is also useful in food preservation, safe cosmetics preparation, medical devices and water treatment.

**Table 6:** The antibacterial activity results of Sr_1−x_Al_2_O_4_:Eu_x_ (x = 0.00 and 0.05 ) phosphor (SA and SAE phosphor) with *Staphylococcus aureus* (*S. aureus*) and *Escherichia coli* (*E. coil*) bacteria.

Bacteria	Antibacterial activity results				
	Phosphor		Control		
	SA	SAE	Al	Sr	Eu
Gram-positive *Staphylococcus aureus* bacteria	No	No	Yes	No	No
Gram negative *Escherichia coli* bacteria	No	No	Yes	No	No

## Conclusion

The Sr_1−x_Al_2_O_4_:Eu_x_ (x = 0.00, 0.01, 0.03, 0.05, 0.07, and 0.09) phosphor was successfully synthesized via urea fuel combustion method. The kind of phase and crystal structure of the sample were confirmed using XRD data, which revealed that it was a single phase with a monoclinic structure and a space group of P 21/n. The FTIR technique was used to chemical bonding behaviour of synthesized phosphor. According to an optical band gap analysis, the sample is a broad band gap material; the value of optical band gap in the current case (SAE 0.05) is 3.78 eV. From Transmission electron microscopy (TEM), the average size found around 6–25 nm. The Image J software was used to compute the fringe width of the HRTEM image. The estimated inter-planner spacing d = 0.222 nm for (231) plan from XRD results is quite close to the observed inter-planner spacing d = 0.22 nm for HRTEM image. The PL emission spectra displayed characteristic glow peaks corresponding to Eu^2+^ in the visible range. When we change the concentration of activator Eu in the host SrAl_2_O_4_ the nature of emission is change accordingly. The Sr_1−x_Al_2_O_4_:Eu_x_ (x = 0.05) phosphor give maximum emission intensity. It can be observed that the Sr_1−x_Al_2_O_4_:Eu_x_ (x = 0.05) phosphor can be regulated as green (0.23, 0.49) emission with the colour temperature 3224 K, CRI 78 and colour purity 60.69%. The use of broad photo emission spectra to obtain direct green emission for green light emitting diodes and other optoelectronic devices could be considered another possibility; the green lights can regulate the circadian rhythm through melatonin. Also the Sr_1−x_Al_2_O_4_:Eu_x_ (x = 0.00 and 0.05) phosphor behaves like eco-materials. Because nano particles of Sr_1−x_Al_2_O_4_:Eu_x_ (SAE 0.05) does not show antibacterial activity. It is applicable as an optical coating agent for making solid state luminescent devices and it is also useful in food preservation, safe cosmetics, medical devices and water treatment.

## Supplementary Information

Supplementary Table 1.

## Data Availability

On the behalf all authors, the corresponding author state that materials described in the manuscript, including all relevant raw data will be freely available to any researcher wishing to use them for non-commercial purposes, without breaching participant confidentiality. The corresponding author also states that the information on where data supporting the results reported in the article can be found, if applicable. When and where applicable, hyperlinks to publicly archived datasets analyzed or generated during the study.
