# Supplementary material for: Investigation of structural, optical, morphological, photoluminescence and antimicrobial properties of SrAl2O4:Eu2+ nanophosphor by using urea fuel combustion method
Source: Sci Rep. 2023 Feb 7;13:2188. doi: 10.1038/s41598-023-29241-4 (PMC9905549; doi:10.1038/s41598-023-29241-4)
Supplement: Supplementary file 1 — Supplementary Table 1. [file 41598_2023_29241_MOESM1_ESM.docx]

**Table :** Structural Parameters- Peak Position (2θ), Miller indices (hkl), Crystallite size D (nm), Dislocation density, Microstrain and Inter-planner spacing d (nm) of Sr_1-x_Al_2_O_4:_ Eu_x_ (x= 0.05) phosphor (SAE 0.05 phosphor).

| **S.N.** | **(hkl)** | **Peak Position** | **FWHM** | **d Spacing** | **Crystallite Size** | Dislocation density | **Microstrain** |
| --- | --- | --- | --- | --- | --- | --- | --- |
|  |  | **(2 θ°)** | **(β°)** | **(nm)** | **D (nm)** | δ × 10^-3^ (nm^-2^) | ε × 10^-3^ |
| 1. | (011) | 19.8230 | 0.3170 | 0.4475 | 25.4357 | 1.5456 | 7.9173 |
| 2. | (120) | 22.5342 | 0.3524 | 0.3942 | 22.9850 | 1.8928 | 7.7186 |
| 3. | (211) | 28.2714 | 0.2907 | 0.3154 | 28.1737 | 1.2598 | 5.0378 |
| 4. | (220) | 29.1082 | 0.2889 | 0.3065 | 28.4021 | 1.2396 | 4.8566 |
| 5. | (211) | 29.7529 | 0.2585 | 0.3000 | 31.7956 | 0.9891 | 4.2463 |
| 6 | (310) | 32.0602 | 0.5303 | 0.2789 | 15.5868 | 4.1160 | 8.0534 |
| 7. | (002) | 34.8833 | 0.3411 | 0.2569 | 24.4119 | 1.6780 | 4.7373 |
| 8. | (231) | 40.5490 | 0.3207 | 0.2222 | 26.4062 | 1.4341 | 3.7882 |
| 9. | (400) | 42.7347 | 0.2997 | 0.2114 | 28.4592 | 1.2346 | 3.3429 |
| 10. | (222) | 45.0707 | 0.2895 | 0.2009 | 29.7007 | 1.1336 | 3.0452 |
| 11. | (240) | 46.2262 | 0.3151 | 0.1962 | 27.4123 | 1.3307 | 3.2213 |
| 12. | (213) | 59.9640 | 0.2626 | 0.1541 | 34.9208 | 0.8200 | 1.9863 |
| 13. | (521) | 62.6387 | 0.3479 | 0.1481 | 26.7299 | 1.3995 | 2.4947 |

From above table and from HRTEM, The homogeneous lattice fringe with estimated inter-planner spacing d= 0.22nm by using TEM data and observed inter-planner spacing d= 0.222 nm for (231) plan from XRD result is highly matched for Sr_1-x_Al_2_O_4_ :Eu_x_ (x= 0.05) phosphor (SAE 0.05)

**Abbreviations**

XRD- X-rays diffraction,

TEM - Transmission electron microscope

SEM- Scanning electron microscope

EDS- Energy dispersive X-ray spectroscopy

PL - Photoluminescence

FWHM - Full width at half maximum

SAED - Selected area electron diffraction pattern

LED - Light emitting diode

HRTEM - High resolution Transmission electron microscope

CIE- Commission international de I’Elcairage

JCPDS - Joint committee on powder diffraction standards

CRI- color-rendering index

CCT- colour correlated temperature

LER- luminous efficacy of radiation

*E. coli- Escherichia coli*

*S. aureus*- *Staphylococcus aureus*

Luria-Bertani broth - LB broth
